# Shear Stress and RBC-NOS Serine1177 Phosphorylation in Humans: A Dose Response

**DOI:** 10.3390/life11010036

**Published:** 2021-01-08

**Authors:** Jarod T. Horobin, Surendran Sabapathy, Lennart Kuck, Michael J. Simmonds

**Affiliations:** 1 Menzies Health Institute Queensland, Griffith University, 4222 Gold Coast, Australia; s.sabapathy@griffith.edu.au (S.S.); lennart.kuck@griffithuni.edu.au (L.K.); m.simmonds@griffith.edu.au (M.J.S.); 2 Biorheology Research Laboratory, Griffith University, 4222 Gold Coast, Australia

**Keywords:** nitric oxide synthase, erythrocytes, mechanotransduction, immunocytochemistry, mechanobiology, biorheology

## Abstract

Red blood cells (RBC) express a nitric oxide synthase isoform (RBC-NOS) that appears dependent on shear stress for Serine1177 phosphorylation. Whether this protein is equally activated by varied shears in the physiological range is less described. Here, we explored RBC-NOS Serine1177 phosphorylation in response to shear stress levels reflective of in vivo conditions. Whole blood samples were exposed to specific magnitudes of shear stress (0.5, 1.5, 4.5, 13.5 Pa) for discrete exposure times (1, 10, 30 min). Thereafter, RBC-NOS Serine1177 phosphorylation was measured utilising immunofluorescence labelling. Shear stress exposure at 0.5, 1.5, and 13.5 Pa significantly increased RBC-NOS Serine1177 phosphorylation following 1 min (*p* < 0.0001); exposure to 4.5 Pa had no effect after 1 min. RBC-NOS Serine1177 phosphorylation was significantly increased following 10 min at each magnitude of shear stress (0.5, 1.5, 13.5 Pa, *p* < 0.0001; 4.5 Pa, *p* = 0.0042). Shear stress exposure for 30 min significantly increased RBC-NOS Serine1177 phosphorylation at 0.5 Pa and 13.5 Pa (*p* < 0.0001). We found that RBC-NOS phosphorylation via shear stress is non-linear and differs for a given magnitude and duration of exposure. This study provides a new understanding of the discrete relation between RBC-NOS and shear stress.

## 1. Introduction

The components of the cardiovascular system (i.e., heart, vessels, and blood) are constantly exposed to flow-induced/-mediated shear stress that stimulates biochemical pathways. Indeed, one of the most important signalling molecules within the cardiovascular system, nitric oxide (NO), may be endogenously produced via shear stress. Nitric oxide is a key mediator of vascular homeostasis and exerts multiple protective functions due to its free radical nature, and the ability of nitrogen to assume a variety of oxidation states [1]. The vasculoprotective functions of NO include antioxidant effects and respiratory control [2] and is well recognised as an important regulator of vessel tone. Circulating NO is thought to be predominantly generated by the enzyme endothelial NO synthase (eNOS), which is expressed within the endothelial layer lining the vasculature: NO is generated when L-arginine binds within the oxygenase domain of NOS and is converted to L-citrulline [3,4]. Although eNOS is well known to be the primary source of NO for regulating vascular tone, the presence of an active eNOS isoform within red blood cells (RBC-NOS) may present an alternate source of NO generation within the vasculature. For patients with a damaged or impaired endothelium (e.g., atherosclerosis patients) [5], this alternative source of NO may be of particular importance for maintaining vessel tone.

Seminal work by Kleinbongard et al. [6] identified that RBC may endogenously produce NO via a local eNOS-like isoform (i.e., RBC-NOS) that shares several characteristics with eNOS. Nitric oxide derived from RBC-NOS may increase RBC deformability via direct S-nitrosylation of cytoskeletal proteins, α- and β-spectrin [7] or contribute to the vascular pool of bioavailable NO. Within the RBC, NO readily binds to deoxyHb which has a high affinity for membrane bound anion exchanger, Band-3, to form iron-nitrosyl-Hb [8]. Jia et al. [9] elucidated that as RBC move into regions of increased PO_2_ (e.g., pulmonary system), NO translocates to the cysteine β93 residue to form SNO-Hb [10]. As RBC traverses the arterial system and PO_2_ once again decreases, NO loses its binding affinity for the cysteine β93 residue of SNO-Hb and is exported out of the cell via Band-3 [11]. Alternatively, NO may also bind with OxyHb to form MetHb and nitrate, and can rapidly react with O_2_ to form nitrite [12,13,14]. The allosteric release of NO from RBC in the form of nitrite has also been suggested to be via Band-3, based on a decrease in plasma nitrite following inhibition of Band-3 [15]. As such, the significance of RBC-NOS should not be understated as highlighted by arguably one of the most common disorders affecting RBC and vascular function, sickle cell disease.

Patients with sickle cell disease present with a higher level of RBC-NOS phosphorylation and increased intracellular nitrite levels when compared to healthy patients [16]. This increased RBC-NOS phosphorylation potentially modulates RBC deformability of these rigid RBC [17]; however, the ability of RBC-NOS to contribute to vascular function via NO is impaired in sickle cell disease patients—possibly due to scavenging by free haemoglobin in plasma which is high in sickle cell disease patients [18]. Nevertheless, given that decreased NO bioavailability within the vasculature has been demonstrated to contribute to cardiovascular pathologies including hypertension, atherosclerosis and angiogenesis-associated disorders [19,20,21], and the ability of RBC-derived NO to potentially contribute to enhanced RBC deformability [7], platelet function [22], and vascular tone [23], elucidating the regulation of RBC-NOS within the vasculature may be of clinical importance.

The enzyme RBC-NOS demonstrates minimal activity for cells at stasis [24] and thus appears to be dependent upon shear stress for activation and production of NO in what appears to be a calcium-/calmodulin-dependent fashion [25,26]. Within endothelial cells, shear stress may activate protein kinase A, and protein kinase B (also known as Akt) pathways via mechanotransduction to increase inositol triphosphate production [27,28]. Subsequently, the regulatory carboxy-terminal tail of eNOS may be displaced via post-translational phosphorylation of the key amino acid, Serine1177, thus relieving repression of NO synthesis by stimulating an influx of electrons within the reductase domain and increasing the Ca^2+^ sensitivity of eNOS [29]. It is not just shear stress that stimulates Akt phosphorylation but also growth factors including VEGF and insulin [30]. As such, it appears eNOS Serine1177 phosphorylation is integral for modulation of enzyme activity. Furthermore, the ability of eNOS Serine1177 phosphorylation to increase Ca^2+^ sensitivity (also referred to as a calcium-independent pathway) in the modulation of NOS activity is of importance for patients receiving calcium channel inhibitors (e.g., patients with high blood pressure/atherosclerosis) where intracellular levels of calcium are likely to be decreased. Similarly, RBC-NOS has been reported by several groups to be phosphorylated at Serine1177 via the phosphoinositide-3 (PI3) kinase/Akt kinase pathway [7,16,31] in response to shear stress and insulin [7]. These findings support research that has demonstrated RBC-NOS Serine1177 phosphorylation may improve RBC deformability, and that calcium channel blockers (which decrease intracellular calcium levels [32]) do not impede RBC deformability in hypertension patients [33]. The resolution of other important aspects of RBC-NOS, however, remains poor. Understanding the tolerance of RBC-NOS to shear stress, for example, would be useful to determine the potential (dys)regulation of this source of NO.

The physiological limits of shear stress within the cardiovascular system have been reported from 0.1 to 0.6 Pa across the venous network, while spanning 1 to 15 Pa within the arterial network [34,35]. Whilst previous studies have examined discrete magnitudes of shear stress on RBC-NOS phosphorylation [23,25], or examined RBC-NOS phosphorylation after being exposed to high-shear environments in either pulsatile or continuous flow [36], the ability to interrogate the effects of this species requires further understanding of the discrete relation between RBC-NOS Serine1177 phosphorylation and shear stress. Indeed, the relationship between RBC-NOS and shear stress has yet to be adequately characterised. The primary aim of the current study was to assess RBC-NOS Serine1177 phosphorylation across a physiologically relevant range of constant shear stresses: previous work suggests no difference between pulsatile and continuous shear stress. The secondary aim of the current study was to identify whether Serine1177 phosphorylation of RBC-NOS is dependent upon the bioavailability of L-arginine via inhibition with Nω-Nitro-L-arginine methyl ester hydrochloride (L-NAME) which competitively binds to the arginine binding site and thus inhibits NO production.

## 2. Material and Methods

### 2.1. Subjects and Sampling

Venous blood was collected from 10 healthy male donors only (age: 28 ± 7 years) given gender differences in haematological, hemorheological, and especially NO-related parameters [37], and anticoagulated with lithium heparin (BD Vacutainer^®^; 158 USP). Written and witnessed informed consent was provided by each participant and the use of blood was consistent with The Code of Ethics of the World Medical Association (Declaration of Helsinki). The experimental procedures were reviewed and approved by the Griffith University Human Research Ethics Committee (HREC GU Ref. 2016/712).

### 2.2. Experimental Overview

A subset of four blood samples were evaluated per subject in the present study which involved three phases. The first phase was measurement of whole blood viscosity to facilitate the calculation of individualised shear stress exposure protocols. The second phase was a “conditioning” period during which discrete blood samples were exposed to a specific magnitude of shear stress (i.e., 0.5, 1.5, 4.5, and 13.5 Pa) for 30 min—the order of which was randomised to ensure no system bias in the data. During each “conditioning” periods, an aliquot of blood was aspirated from the shearing device following 1, 10, and 30 min to allow for the examination of exposure duration on the dependent variables; the process of ceasing shearing and collecting each sample took <30 s in total. The third phase was fixation of blood samples immediately following the aspiration from the shearing device for preservation of RBC-NOS phosphorylation. The durations of exposure were chosen based on previous works that have investigated Serine1177 phosphorylation of eNOS, and mechanical changes to RBC following exposure to shear stress [25,27,38]. Basal levels of RBC-NOS phosphorylation were examined by using an aliquot of RBC that had not been previously sheared (i.e., control); these samples were collected and fixed in the same manner as the experimental data.

To evaluate the second Aim and provide further understanding of the key characteristics of RBC-NOS, a second study (*n* = 5) was conducted to assess the inhibitory effect of L-NAME on RBC-NOS phosphorylation. This sub-study involved the evaluation of four blood samples: (1) RBC exposed to a shear stress conditioning of 13.5 Pa for 10 min; (2) RBC pre-incubated with 5 mM L-NAME (5 mM; N5751, Sigma-Aldrich; St. Louis, MO, USA) for 1 h at room temperature and then exposed to a shear stress conditioning of 13.5 Pa for 10 min; (3) RBC with no prior exposure to shear stress; and (4) RBC pre-incubated with 5 mM L-NAME for 1 h at room temperature with no prior exposure to shear stress.

### 2.3. Exposure of Red Blood Cells to Shear Stress

Viscosity (η) of blood at native haematocrit was measured using a calibrated rotational cone-plate viscometer operated at 37 °C (0.5 DVII+ with CPE40 spindle, Brookfield Engineering Labs; Middleboro, MA, USA). Thereafter, a fresh blood subset was loaded into the shearing system for the application of shear stress: the shearing system is principally employed for the measurement of RBC deformability from a low haematocrit sample of RBC suspended in a Newtonian viscous fluid. As whole blood samples were used, however, the measurement of deformability was not possible in this study. The shearing system (LORRCA MaxSis, Mechatronics; Zwaag, Hoorn, The Netherlands) consists of two concentric cylinders; the outer cylinder is temperature-regulated at 37 °C and is rotated at discrete speeds to apply a shear rate to the fluid between the two cylinders. Thus, for the experimental protocol, the shear stress could be calculated given that the viscosity of blood was measured, and the shear rate ($\dot{\gamma }$) applied to the gap of the shearing system could be quantified. First, to calculate the shear rate required to apply the desired magnitude of shear stress for a known viscosity solution, the following equation described by Hardeman, Goedhart [39] was used:

(1) $$\dot{\gamma }=2\times \frac{2\pi }{60}\;\frac{r_{1}\times r_{2}}{r_{2}^{2}-r_{1}^{2}}\times N$$

where *r*_1_ and *r*_2_ = radius of the inner and outer cylinder, respectively; *N* = rev/min of the outer cylinder. The shear stress was then calculated using the equation:

(2) $$\tau =\eta \;\times \;\dot{\gamma }$$

Subsequently, the rotational speed of the outer cylinder was set to obtain the pertinent shear rate for the discrete magnitude of shear stress to be applied. A fresh whole blood sample (1200 µL) was utilised for each discrete assessment. The shearing system was then operated for 1 min, after which shearing ceased for 10 s to allow for a small portion (300 µL) of RBC suspension to be collected and immediately fixed with 4% paraformaldehyde. Application of shear was immediately recommenced, and the experimental protocol resumed for a further 9 min (i.e., 10 min of accumulated shear exposure). The shearing system was then paused a second time, to allow for another small portion (300 µL) of RBC suspension to be collected and fixed. Application of shear recommenced once again and the experimental protocol resumed for a further 20 min (i.e., a total of 30 min shear exposure), before a final sample of RBC suspension was collected and fixed. All samples collected during this protocol were subsequently analysed for RBC-NOS Serine1177 phosphorylation. Note that for the secondary study (i.e., evaluation of L-NAME on RBC-NOS phosphorylation) all sheared samples were exposed to shear for 10 min continuously without ceasing after 1 min.

### 2.4. Red Blood Cell Nitric Oxide Synthase Serine1177 Phosphorylation

**Figure 1:**
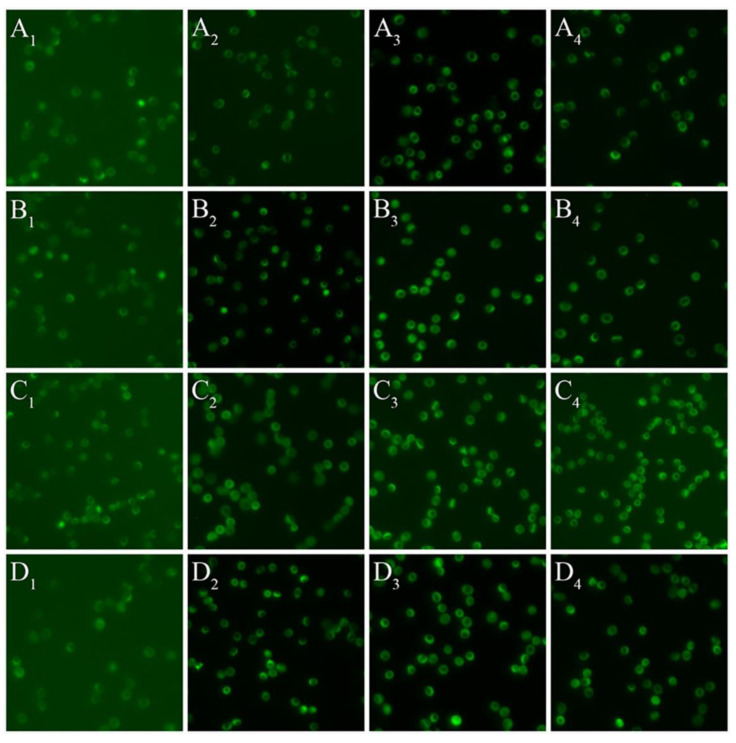
Fluorescence images of activated red blood cell-derived nitric oxide synthase following exposure to shear stress exposed to discrete magnitudes ((**A**): 0.5 Pa, (**B**): 1.5 Pa, (**C**): 4.5 Pa, (**D**): 13.5 Pa) and durations (subscripts 1–4; representing 0, 1, 10 and 30 min, respectively).

The quantification of RBC-NOS Serine1177 phosphorylation was performed utilising an immunohistochemistry method adapted from published methods [7,31,40] with a fluorescence detection method developed in-house. Suspensions of RBC collected during the shearing protocol were fixed with 4% paraformaldehyde in tris-buffered saline (TBS, pH 7.6) at a 1:1 ratio for 20 min. Following fixation, samples were smeared onto a glass slide and a wax border was made around each blood smear to separate between the “test” area and negative control area using a Super PAP Pen (Catalogue No. 008899; ThermoFisher Scientific; Waltham, MA, USA). The blood smears were permeabilised with 0.1% trypsin and then incubated with a blocking buffer (3% milk powder in 0.1 M TBS with 0.001% Tween-20) for 30 min: this antigen retrieval step was performed to ensure the antigenicity of phosphorylated RBC-NOS was not reduced via formation of hydroxyl-methylene bridges [41,42]. The test area of each slide was then incubated with anti-phospho eNOS (Ser1177; Merck Millipore; Burlington, MA, USA) in antibody diluent (TBS with 0.3% milk powder and 0.001% Tween-20, 1:200), while the control area was incubated with antibody diluent in the absence of the primary antibody. The increased dilution of milk-powder for the primary antibody incubation step was employed to minimise non-specific binding [43]. Afterward, the blood smears were incubated with a secondary blocking buffer (3% goat serum in 0.1 M TBS) for 1 h, followed by incubation with a goat anti-rabbit IgG secondary antibody (Vector Laboratories; Burlingame, CA, USA) in antibody diluent for 1 h. A transition to goat serum (i.e., the species in which the secondary antibody was raised) was ventured to minimise non-specific binding and decrease background signal. The blood smears were then washed with TBS several times, dehydrated with a graded series of alcohols (70, 90, and 100%), cleared with xylene (534056, Sigma-Aldrich; St. Louis, MO, USA), and then mounted with a purpose medium (Entellan^®^ new; Merck Millipore; Burlington, MA, USA) and glass coverslip. Finally, smears were examined with a fluorescence objective (LUCPLFLN 40X/0.60) attached to an inverted microscope (IX73, Olympus Corp., Tokyo, Japan) coupled to a CMOS camera (optiMOS™ sCMOS; QImaging; Surrey, BC, Canada). Digital photos of the test area and negative control area were analysed using Image J software (National Institutes of Health, Bethesda, MD, USA), with the intensity of immunofluorescence representing the amount of RBC-NOS Serine1177 phosphorylation. The exposure time and gain settings for all images depicted in Figure 1 were set to be identical to minimise bias in interpretation of fluorescence intensity; however, this at times increased background “noise” (intensity of background fluorescence intensity) due to the large range of intensity between samples. The differences in these background intensities were accounted for, however, as described below in Data Analysis.

### 2.5. Data Analysis

Initially, immunofluorescence intensity of both the test area and the control were quantified as the RBC grey value minus the mean background grey value, which was detected at a cell-free area of the slide; at least six cell-free regions were used to calculate the background value on each slide. Thereafter, the corrected total cell fluorescence (CTCF) for each cell was determined by subtraction of the immunofluorescence intensity of the control area from the immunofluorescence of the test area (≥50 cells) as has been previously reported [16,44].

### 2.6. Statistical Analysis

The D’Agostino and Pearson test of normality was used to assess normality of the CTCF data. Subsequently, the Kruskal–Wallis test was used to determine whether significant differences in the mean CTCF existed. Dunn’s multiple comparisons test was applied when examining multiple comparisons between durations of shear stress exposure for a given magnitude (Prism 7.04, GraphPad Software Inc.; San Diego, CA, USA). For assessment between samples pre-incubated with L-NAME and samples untreated, a two-way ANOVA was used to determine whether significant differences in the mean CTCF existed. The Bonferroni post hoc test was then employed when examining multiple comparisons (Prism 7.04, GraphPad Software Inc.; San Diego, CA, USA).

## 3. Results

### 3.1. Whole Blood Viscosity

Prior to accurate application of shear to samples, discrete blood viscosity was required to be measured to facilitate shear stress calculations; consequently, each sample’s viscosity was determined across a dynamic range of shear rates. The shear stress was calculated using a relation between governed shear rate and measured viscosity. Thereafter, a matrix of shear stresses and respective shear rates was generated to achieve the desired shear stresses of 0.5, 1.5, 4.5, and 13.5 Pa. To obtain a shear stress magnitude of 0.5 Pa, a shear rate of 122 ± 17 s^−1^ was applied to whole blood samples. Likewise, a shear rate of 300 ± 14, 900 ± 41, and 2712 ± 116 s^−1^ was applied to whole blood samples to obtain shear stress magnitudes of 1.5, 4.5, and 13.5 Pa, respectively.

### 3.2. Red Blood Cell Nitric Oxide Synthase Serine1177 Phosphorylation

**Figure 2:**
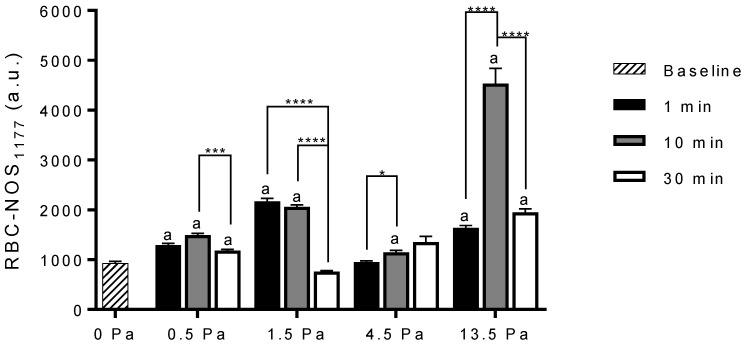
Corrected total cell fluorescence of Serine1177 phosphorylated RBC-NOS at baseline (0 Pa) and Figure 1. 10, and 30 min, at shear stress magnitudes of 0.5, 1.5, 4.5, and 13.5 Pa. Data are presented as mean ± SEM. * *p* < 0.05, *** *p* < 0.001 significant difference, **** *p* < 0.0001 significant difference. ^a^ significant difference from RBC-NOS Serine1177 phosphorylation before shear stress. With *P* values presented in text.

The mean RBC-NOS Serine1177 phosphorylation for blood samples exposed to shear stress stimulation at 0.5, 1.5, 4.5, and 13.5 Pa for durations of 1, 10, and 30 min is presented in Figure 1 and Figure 2. There was a significant increase in RBC-NOS Serine1177 phosphorylation following exposure to 0.5 Pa for 1 min (1294 ± 33 a.u.; *p* < 0.0001), 10 min (1494 ± 35 a.u.; *p* < 0.0001), and 30 min (1184 ± 24 a.u.; *p* < 0.0001), when compared with baseline (934 ± 33 a.u.; 0 Pa). There was a significant decrease, however, in RBC-NOS Serine1177 phosphorylation following 30 min exposure to 0.5 Pa when compared to an exposure duration of 10 min (*p* = 0.0004).

The mean Serine1177 phosphorylation of RBC-NOS was significantly increased following shear exposure to 1.5 Pa for 1 min (2173 ± 57 a.u.; *p* < 0.0001) and 10 min (2063 ± 39 a.u.; *p* < 0.0001), when compared to baseline. No significant difference was observed following exposure to 1.5 Pa for 30 min (764 ± 20 a.u.). Exposure to 1.5 Pa for 1 min induced the greatest response, as RBC-NOS Serine1177 phosphorylation significantly decreased when compared to an exposure duration of 30 min (*p* < 0.0001). Moreover, RBC-NOS Serine1177 phosphorylation was significantly decreased following 30 min shear stress exposure at 1.5 Pa when compared to an exposure duration of 10 min (*p* < 0.0001).

Exposure to a shear stress magnitude of 4.5 Pa for 1 min (952 ± 25 a.u.) did not induce a significant change in RBC-NOS Serine1177 phosphorylation when compared to baseline; however, there was a significant increase following 10 min (1146 ± 42 a.u.; *p* = 0.0042). Indeed, the Serine1177 phosphorylation of RBC-NOS after 10 min exposure at 4.5 Pa was significantly greater when compared to an exposure duration of 1 min (*p* = 0.0275). Following exposure to 4.5 Pa for 30 min, there was no significant difference in RBC-NOS Serine1177 phosphorylation when compared to baseline (1351 ± 116 a.u.).

The mean RBC-NOS Serine1177 phosphorylation was significantly increased following exposure to 13.5 Pa for 1 min (1635 ± 51 a.u.; *p* < 0.0001), 10 min (4534 ± 305 a.u.; *p* < 0.0001) and 30 min (1953 ± 70 a.u.; *p* < 0.0001) when compared to baseline. Between exposures of 1 and 10 min there was a significant increase in the mean RBC-NOS Serine1177 phosphorylation (*p* < 0.0001). After peaking at 10 min of exposure to 13.5 Pa, RBC-NOS Serine1177 phosphorylation significantly decreased after 30 min (*p* < 0.0001), albeit remaining elevated compared with baseline.

**Figure 3:**
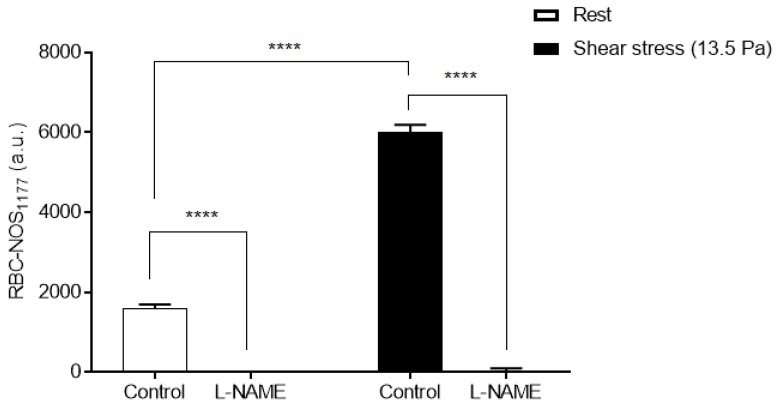
Corrected total cell fluorescence of Serine1177 phosphorylated RBC-NOS following exposure to shear stress of 13.5 Pa for 10 min and at rest in the absence (untreated) or presence of L-NAME. RBC-NOS Serine1177 phosphorylation before shear stress is presented as white bars, and samples exposed to 13.5 Pa for 10 min are presented as black bars. Data are presented as mean ± SEM, *n* = 5. **** *p* < 0.0001 significant difference.

The mean RBC-NOS phosphorylation for cells exposed to a shear stress magnitude of 13.5 Pa for 10 min (without a pause at 1 min as performed in the previous experiment), and cells that were unsheared (i.e., rest) in the absence (untreated) or presence of L-NAME are presented in Figure 3. There was a significant increase in RBC-NOS phosphorylation following exposure to 13.5 Pa for 10 min when compared to static non-sheared samples (*p* < 0.0001). Samples that were previously sheared and also those unsheared and pre-incubated with L-NAME had significantly decreased fluorescence when compared to respective Control samples (*p* < 0.0001). No significant difference in fluorescence of samples pre-incubated with L-NAME was observed between rest samples and sheared samples.

## 4. Discussion

The aim of the present study was to examine changes in RBC-NOS Serine1177 phosphorylation stimulated by discrete magnitudes and durations of shear stress exposure. The principal finding was that the stimulation of RBC-NOS via shear stress is dependent upon both shear stress magnitude and duration in a non-linear manner. Low shear (i.e., 0.5 Pa) significantly increased RBC-NOS Serine1177 phosphorylation following exposure at all durations when compared to baseline; however, the magnitude of RBC-NOS Serine1177 phosphorylation was significantly decreased at 30 min exposure when compared to 10 min exposure (Figure 2). Phosphorylation of RBC-NOS Serine1177 at 1.5 Pa was induced in a rapid fashion but declined rapidly with continued exposure (e.g., 30 min exposure was significantly decreased compared to 10 min exposure). A shear stress magnitude of 4.5 Pa induced the least amount of RBC-NOS Serine1177 phosphorylation: a significant difference in RBC-NOS Serine1177 phosphorylation was observed only following 10 min when compared to baseline. Finally, a significant increase in RBC-NOS Serine1177 phosphorylation was observed following high shear exposure (i.e., 13.5 Pa) for all durations when compared to baseline with the greatest magnitude of RBC-NOS Serine1177 phosphorylation observed following 10 min exposure.

The phosphorylation of RBC-NOS was significantly increased following exposure to a shear stress magnitude of 0.5 Pa for all durations of exposure when compared to baseline; however, a significant decrease was identified after 30 min when compared to durations of 10 min. These data suggest RBC-NOS reaches maximal phosphorylation relative to 0.5 Pa after 10 min. To the best of our knowledge, no previous in vitro or in vivo studies have directly examined RBC-NOS or eNOS phosphorylation at 0.5 Pa. However, Dimmeler et al. [45] previously observed via eNOS immunoprecipitation and phosphoamino acid analysis a significant increase in Akt phosphorylation following exposure to a shear stress magnitude of 5 dyn/cm^2^ (i.e., 0.5 Pa), and determined Akt phosphorylation significantly increased eNOS phosphorylation. Dimmeler et al. [45] observed a maximal response in Akt phosphorylation following 1–2 h which suggests the time to maximal eNOS phosphorylation is likewise greater than the maximal response observed for RBC-NOS in the current study. Indeed, an in silico study by Koo et al. [46] determined that the time to maximal eNOS phosphorylation following exposure to 0.5 Pa is approximately 30 min. The current study indicates that RBC-NOS exposed to 0.5 Pa exhibits an ability to reach maximum enzymatic activation that is expedited when compared to eNOS. It is possible this difference is the result of substrate availability. As eNOS is membrane bound, Shin et al. [47] determined that endothelial cells are reliant on extracellular (i.e., plasma) bioavailability of L-arginine: circulating plasma concentration of L-arginine is approximately 50–100 µM. In contrast, RBC-NOS which is found within the intracellular and membrane compartments [6], may rapidly uptake 200 µM L-arginine, in approximately 10 min [48] which stimulates NOS phosphorylation through the PI3 kinase/Akt kinase pathway [49,50].

The present study demonstrates that exposure to shear stress at 1.5 Pa significantly increased RBC-NOS phosphorylation after 1 min and 10 min. These data are congruent with the findings by Boo et al. [27] (observed via Immunoblotting), where eNOS activity, as indicated by Serine1177 phosphorylation, was stimulated via shear stress after 2 min at 1.5 Pa. The present data and the data from Boo et al. [27] are not directly comparable, however, due to differences in detecting NOS phosphorylation. Continued exposure to a shear magnitude of 1.5 Pa in the present study saw a decline in RBC-NOS phosphorylation following 10, and 30 min when compared to RBC-NOS phosphorylation at 1 min. No previous studies have directly investigated the effects of a shear stress exposure at 1.5 Pa on RBC-NOS phosphorylation; however, the decline in RBC-NOS phosphorylation following 1 min shear exposure at 1.5 Pa may be explained by negative feedback common to eNOS isotypes [51]. It has been previously demonstrated within endothelial cells that eNOS activity may be decreased via shear-mediated processes that produce secondary species, such as peroxynitrite, that may lead to oxidation of the eNOS enzyme [52,53]. It is well established that NO readily binds to superoxide to form the oxidant, peroxynitrite, and that peroxynitrite readily oxidises biomolecules, and may form nitrated adducts [54,55,56]. Given the significant increase in RBC-NOS phosphorylation following 1 min, it is likely that intracellular NO bioavailability was increased at this time-point. Consequently, it is plausible that RBC-NOS activity (due to phosphorylation) may have been auto-regulated by the accumulation of intracellular NO to minimise oxidative stress induced by increased RBC-NOS enzymatic activity. Indeed, the prolonged shears in the present study may have led to an imbalance in normal redox state of the cells, that experience fluctuating but highly intermittent shears during their passage of the circulatory system.

The phosphorylation of RBC-NOS following exposure to 4.5 Pa was highly dependent upon exposure time; no changes were observed after 1 and 30 min of exposure, although was significantly increased after 10 min exposure. Although no previous studies have investigated the effects of a shear stress magnitude of 4.5 Pa on eNOS phosphorylation, these data are consistent with the emergent hypothesis that prolonged exposure to higher magnitudes of shear stress may lead to auto-regulation of RBC-NOS enzymatic activity via negative feedback induced by accumulated intracellular NO. A limitation of the present study, however, is that NO was not measured directly due to the methodology employed, thus no definitive conclusion can be made at this time.

The present study observed a significant increase in RBC-NOS phosphorylation following exposure to a shear stress magnitude of 13.5 Pa for all durations when compared to baseline. The shear stress magnitude of 13.5 Pa utilised in the present study represents the upper limits of shear stress observed within the circulatory system of healthy individuals [34]. It is plausible this is due to the functional requirements of RBC needing to maximally deform when traversing blood vessels, and the release of NO for propagation of microvascular control that may aid in effective oxygenation [57,58]. It is within the microcirculation that RBC are exposed to the upper limits of shear stress, as well as an oxygen gradient where ~50–65% of haemoglobin-bound oxygen is released to venous exchange [59,60,61]. Stamler et al. [10] proposed NO may be released from haemoglobin in areas with low partial pressure of oxygen to regulate local blood flow. However, oxygen demand and oxygen levels are often spatially heterogeneous in tissues and as such, upstream communication to the arteriole is required from the downstream capillaries for effective oxygenation [58]. Therefore, given the present results, it is plausible that microvascular control is not only a function of oxygenation state, nitric oxide-mediated vasodilation, and conducted responses propagated upstream along the vasculature, but also mechanotransduction. In other words, a critical threshold may exist above 4.5 Pa and/or equal to 13.5 Pa for the phosphorylation of RBC-NOS and subsequent production and release of RBC-derived NO for maintenance of vascular control. The exact mechanism for sustained activation of RBC-NOS at high shear was not investigated in this study; however, the sensitisation of RBC-NOS to calmodulin may be enhanced at high shear via crosstalk of associated proteins in a manner similar to eNOS [62,63,64], thus overcoming any auto-regulation of enzymatic phosphorylation. Nevertheless, limitations of the current study necessitate further research into the effects of shear stress on RBC-NOS protein-protein interactions.

In the present study, L-NAME completely removed the fluorescence signal of RBC-NOS under static conditions (e.g., rest) and when exposed to 13.5 Pa of shear stress constant for 10 min (Figure 3). L-NAME competes with the substrate L-arginine for preferential binding at the arginine-binding site within the oxygenase domain of NOS to inhibit NOS activity [65]. Given that RBC-NOS Serine1177 phosphorylation occurs within the reductase domain, it was not clear whether L-NAME would affect RBC-NOS Serine1177 phosphorylation per se. Nevertheless, several studies have demonstrated that L-arginine plays a role in the phosphorylation and activation of the PI3 kinase/Akt kinase pathway [49,50]. Given the present findings, it is plausible that the specific NOS inhibitor, L-NAME, may also indirectly inhibit NOS phosphorylation via inhibition of upstream NOS regulatory proteins (e.g., PI3/Akt kinase), at least using the concentration employed in the present study. However, the measurement of PI3 kinase phosphorylation/Akt activity, and enzymatic productivity (i.e., NO concentration), was not employed in the current study, precluding definitive analysis of upstream pathways associated with RBC-NOS phosphorylation and RBC-NOS-derived NO production following shear exposure.

## 5. Conclusions

The current study investigated the effect of shear stress exposure on RBC-NOS Serine1177 phosphorylation within the physiological range and identified a non-linear response. The time required to reach maximal RBC-NOS Serine1177 phosphorylation differed between magnitudes of shear stress. It is plausible this is due to interactions with other modulators of NOS such as Threonine495 which has been shown to coordinate eNOS activity with Serine1177 [66,67,68,69]; however, further studies are required. The observed dose-response of RBC-NOS is consistent with the effects of shear stress on RBC in vivo. Further, it was confirmed that L-NAME inhibits shear-mediated increase in RBC-NOS phosphorylation.

## Data Availability

The data that support the findings of this study are available from the corresponding author, J.T.H., upon reasonable request.
